# Relaxin Positively Influences Ischemia—Reperfusion Injury in Solid Organ Transplantation: A Comprehensive Review

**DOI:** 10.3390/ijms21020631

**Published:** 2020-01-17

**Authors:** Lina Jakubauskiene, Matas Jakubauskas, Bettina Leber, Kestutis Strupas, Philipp Stiegler, Peter Schemmer

**Affiliations:** 1 General, Visceral and Transplant Surgery, Department of Surgery, Medical University of Graz, Auenbruggerpl. 2, 8036 Graz, Austria; morozovaite.lina@gmail.com (L.J.); matasjakub@gmail.com (M.J.); bettina.leber@medunigraz.at (B.L.); peter.schemmer@medunigraz.at (P.S.); 2 Faculty of Medicine, Vilnius University, 03101 Vilnius, Lithuania; kestutis.strupas@santa.lt

**Keywords:** liver transplantation, kidney transplantation, heart transplantation, lung transplantation, relaxin, serelaxin, review

## Abstract

In recent decades, solid organ transplantation (SOT) has increased the survival and quality of life for patients with end-stage organ failure by providing a potentially long-term treatment option. Although the availability of organs for transplantation has increased throughout the years, the demand greatly outweighs the supply. One possible solution for this problem is to extend the potential donor pool by using extended criteria donors. However, organs from such donors are more prone to ischemia reperfusion injury (IRI) resulting in higher rates of delayed graft function, acute and chronic graft rejection and worse overall SOT outcomes. This can be overcome by further investigating donor preconditioning strategies, graft perfusion and storage and by finding novel therapeutic agents that could reduce IRI. relaxin (RLX) is a peptide hormone with antifibrotic, antioxidant, anti-inflammatory and cytoprotective properties. The main research until now focused on heart failure; however, several preclinical studies showed its potentials for reducing IRI in SOT. The aim of this comprehensive review is to overview currently available literature on the possible role of RLX in reducing IRI and its positive impact on SOT.

## 1. Introduction

In recent decades, solid organ transplantation (SOT) has increased the survival and quality of life for patients with end-stage organ failure by providing a potentially long-term treatment option [1,2,3]. Nowadays, SOT is not only performed as a lifesaving procedure but also purely for quality of life enhancement measures as the examples of uterus, penis, face or limb transplantation show [4,5,6,7]. This evolution of SOT is possible due to technical and pharmacological advances and standardization of procedures. Although the availability of organs for transplantation has increased throughout the years, the demand greatly outweighs the supply. Kidney, liver, heart and lung remain the most demanded organs, which account for more than 98% of all SOT. According to the Organ Procurement and Transplantation Network data, in 2018, in the United States, there were 36,529 SOTs. Unfortunately, this number is inadequate as the waiting list currently has around 113,000 of possible recipients. The same year over 6000 patients died waiting for a transplant. In 2018, in the Eurotransplant member states, 7350 SOTs were performed; however, there were 1896 patients who died or became unfit while waiting for SOT. Several authors suggest organ shortage to be the biggest challenge in transplant medicine [8,9]. One possible solution for this problem is to extend the potential donor pool by using extended criteria donors (ECD) [10]. As these donors are older and have more comorbidities, organs are more prone to ischemia reperfusion injury (IRI) resulting in higher rates of delayed graft function, acute and chronic graft rejection and worse overall SOT outcomes [11,12,13,14,15]. There is a huge scientific effort to find ways in reducing IRI in order to increase the number of suitable organs procured from ECD. This can be achieved by further investigating donor preconditioning strategies, graft perfusion and storage and by finding novel therapeutic agents that could reduce IRI [16,17,18,19,20]. Relaxin (RLX) is a peptide hormone, which was first isolated from the ovaries and named by Dr. Frederick Hisaw in 1926 after experiments with guinea pigs showed RLX ability to relax the pubic ligament [21]. RLX belongs to the insulin family of structurally related molecules. The majority of circulating RLX in primates is encoded by the RLN2 gene and has a high affinity to cognate receptor relaxin family peptide receptor 1 (RXFP1) [22,23]. These receptors are scattered throughout the body and are found not only in the reproductive organs but also in kidney, lung, heart, liver, brain, arteries and blood cells [24]. Although for a long time RLX was considered strictly as a pregnancy hormone, several researchers demonstrated its antifibrotic, antioxidant, anti-inflammatory and cytoprotective actions and the role in hemodynamic changes [25,26,27,28]. These newly discovered effects prompted to investigate RLX as a pharmacotherapeutic agent in preclinical and even clinical settings. The main research until now focused on the heart failure; however, several preclinical studies showed its potential for reducing IRI in SOT (Table 1).

The aim of this comprehensive review is to overview currently available literature on the possible role of RLX in reducing IRI and its positive impact on SOT.

## 2. Materials and Methods

The literature search was performed in PubMed, Web of Science, EMBASE and ClinicalTrials.gov online databases. The following combination of Medical Subject Headings (MeSH) and keywords with the employment of “*AND*” or “*OR*” Boolean operators were used:

“Organ Transplantation” OR “Transplantation” OR “Organ transplant” OR “liver transplantation” OR “uterus transplantation” OR “kidney transplantation” OR “heart transplantation” OR “lung transplantation” OR “pancreas transplantation” OR “Organ Preservation Solutions” OR “Perfusion” OR “Perfusion machine” OR “Reperfusion Injury” OR “Ischemia Reperfusion Injury” OR “Cold Storage” OR “Cold ischemia” OR “hypoxia-reoxygenation” OR “reperfusion” OR “ischemia” OR “ischaemia” OR “ischemic reperfusion” OR “ischaemic reperfusion” AND “Relaxin” OR “Serelaxin” OR “Recombinant human relaxin”.

Database specific search strategies are provided as a Supplementary Materials. The search was restricted to English language only without a time limitation. Most recent search was performed on 20 October 2019.

At least two researchers reviewed the abstracts for the inclusion. After relevant abstracts were identified, full-text articles were retrieved and re-reviewed. Reference lists from selected studies were examined, and relevant articles were also included. A flowchart of the literature search according to the PRISMA guideline is provided as a Supplementary Materials [29].

## 3. Comprehensive Review

**Figure 1:**
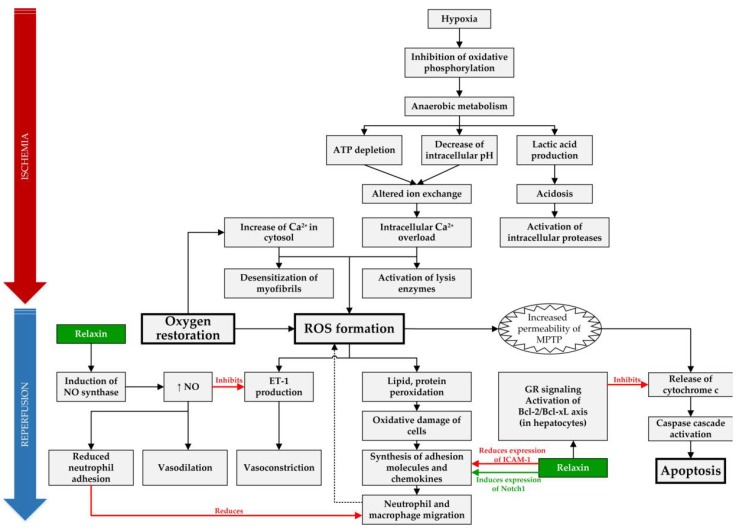
Overview of targets of RLX in IRI. In IRI setting, RLX has anti-inflammatory effect by reducing expression of intracellular adhesion molecule 1 (ICAM-1), inducing expression of Notch1 in macrophages and by reducing neutrophil adhesion through increased synthesis of nitric oxide (NO) [30,36]. Furthermore, it acts as an anti-apoptotic agent in hepatocytes by binding to the glucocorticoid receptors (GR) and inhibiting release of cytochrome c from mitochondria [31]. Lastly, RLX causes vasodilation through increased NO production, which acts as a vasodilator and reduces vasoconstriction by inhibiting endothelin 1 (ET-1) production [38]. (ROS—reactive oxygen species, MPTP—mitochondrial permeability transition pore).

A total of 14 articles examined the possible role of RLX in SOT and are summarized in Table 1, and the molecular mechanisms, which can be targeted and positively influenced by RLX during IRI, are compiled in Figure 1.

### 3.1. Liver Transplantation (LTx)

Numbers of patients waiting for LTx are increasing continuously resulting in a constant enlargement of the gap between organ need and organ availability. Therefore, new techniques to expand the donor pool are coming to clinical practice. Among others, living donor LTx, splitting grafts, the use of machine perfusion or donors who are older or after cardiac death aim to increase the available donor organs [44]. As the transplantations from ECD is rising, and it is known that these organs are more prone to IRI, research in reducing IRI is mandatory [11]. A total of 5 publications analyzing RLX potency in liver were identified. 3 publications by Boehnert et al. evaluated the role of RLX in a perfused rat liver model [32,33,34]. In the first experimental model, University of Wisconsin (UW) solution was used for preservation and the reperfusion was performed with a hemoglobin free solution, additional doses of 32 ng/mL and 64 ng/mL of RLX were added, respectively. Liver ischemia was induced for 3.5 h applying either 20 °C (warm) or 4 °C (cold) preservation conditions [34]. In the second experimental model, liver ischemia was induced for 5 h, and instead of UW, the HTK solution was used with 64 ng/mL of RLX in the preservation solution [32,33]. The authors concluded that RLX had a protective effect against IRI, which was indicated by reduced levels of malondialdehyde (MDA) and myeloperoxidase (MPO) and by the increased scattered light intensity (indicates increased oxygen supply).

The only SOT model examining the role of RLX in the liver was an orthotopic LTx model in mice [30,31]. The liver was stored in 4 °C UW solution for 18 h before implantation, and 5 µg/kg RLX was injected intravenously at the time of reperfusion. RLX significantly improved overall survival rate, decreased the number of apoptotic cells, attenuated sinusoidal congestion, edema/vacuolization and hepatocellular necrosis. However, the main finding of this study was that RLX acts not through its primary cognate receptor RXFP1, but through hepatocyte glucocorticoid receptors. Moreover, Kageyama et al. conducted an additional study using the same protocol and discovered that RLX in liver acts by inducing the macrophage Notch 1 signaling pathway, which is involved in cell fate decision, proliferation, differentiation and regeneration [30,45].

Overall, RLX shows promising results in LTx models, therefore it may be a promising substance to be tested in clinical LTx studies.

### 3.2. Kidney Transplantation (KTx)

According to Global Observatory on Donation and Transplantation (GODT) data, a total of 75,664 KTx were performed worldwide in 2018, retaining it the most transplantable solid organ. Although KTx from living donors are performed quite often, the majority of kidneys are transplanted from deceased donors, which are known to be more prone to IRI [46]. It has been shown that IRI in kidneys is one of the major causes of delayed graft function, chronic graft dysfunction and graft rejection [47].

There are no experimental KTx models using RLX as a protective agent; however, two publications analyzing the role of RLX in reducing kidney IRI in rats [35,36]. In a study by Yoshida et al. an osmotic minipump was used to administer 500 ng/h of RLX for 24 h starting 2 h before ischemia. Another experiment was described in the same publication in which the RLX was infused via the same osmotic minipumps just at the time of reperfusion. The authors found that RLX improves renal function after reperfusion; it also ameliorates the histological damage to the kidney and has an anti- apoptotic effect. Interestingly, staining of RXFP1 showed that damaged cells did not express these receptors, suggesting that pretreatment with RLX should be more effective. However, this idea contradicts the result, which showed increased kidney function when RLX was administered at the time of reperfusion compared to the pretreatment group. Another study by Collino et al. used a similar IR model in rats. Rats were subjected to 1 h of ischemia followed by 6 h of reperfusion. In the experimental group, RLX was administered at the dose of 5 μg/kg intravenously at the beginning of reperfusion and again 3 h after reperfusion. The findings were identical to the previously mentioned study and showed that RLX improved renal function after IR and also ameliorated the histologically verified damage to the kidney. Additionally, RLX reduced local leukocyte recruitment and oxidative stress. None of the above mentioned studies investigated the exact signaling mechanisms of RLX in renal cells; however, they provide data suggesting the involvement of the nitric oxide pathway [35,36].

Although there are some methodological differences between these two studies concerning RLX administration time or duration of reperfusion, both studies concluded that RLX may have beneficial effect in reducing kidney IRI.

### 3.3. Lung Transplantation (LuTx)

First successful LuTx was performed by James Hardy in 1963 [48]. Since then, more than 69,000 procedures have been performed worldwide with the median survival reaching 6.7 years [49]. Although the survival almost doubled in the last 30 years, efforts are necessary to further improve outcome as the rate of primary graft dysfunction after LuTx can reach 30% [50]. This is mostly due to the IRI endured during transplantation [51]. Some authors agree that methods to reduce IRI are deeply needed to improve both short- and long-term outcomes after LuTx [52].

Currently, there are no studies investigating the effect of RLX in experimental LuTx models. Overall, there are only two publications about the potential role of RLX in protecting lungs from IRI by Alexiou et al. [37,38]. In the first study, an isolated lung perfusion model was described using Krebs–Henseleit solution containing 5 nmol/L of RLX. Lungs were exposed to 60 m of ischemia and 60 m of reperfusion [37]. The results of this study showed marked reduction of biochemical and morphological markers of pulmonary injury and reduced activity of neutrophil elastase (NE) and MPO. In addition, the production of MDA also was decreased, as well as production of endothelin-1 (ET-1) from endothelial cells was inhibited by adding RLX to the perfusate. The second study was similar, except the ischemia and reperfusion times were prolonged to 90 m [38]. This study was mainly aimed to determine pathways through which RLX acts in the lungs. By adding various inhibitors to the perfusate, authors discovered RLX protective effect via early and moderate inducible nitric oxide synthase (iNOS) induction, dependent on balanced extracellular signal-regulated kinase-1/2 (ERK-1/2) and phosphatidylinositol-3 kinase (PI3K) stimulation. This experiment also showed that RLX does not affect IRI in lungs if it is applied only during ischemia or only during the reperfusion cycles. Although there is evidence that RLX can reduce IRI in an isolated rat lung model, there are no studies using RLX in an experimental LuTx model. Further investigation is needed in order to better understand the true effect of RLX in LuTx.

### 3.4. Heart Transplantation (HTx)

World’s first HTx was performed by Christiaan Barnard in 1967. Although the results after the first transplantations were poor, the development of modern immunosuppression therapy led to the median survival of around 15 years nowadays [53]. HTx remains crucial in medicine as it is the gold standard treatment in end-stage heart failure [54]. However, the demand of donors exceeds the available supply, requiring new strategies to overcome organ shortage [55]. Extension of the donor pool can be achieved by using ECD organs including organs with prolonged ischemia time or donation after circulatory death. Such grafts are more susceptible to IRI which translates to higher primary graft dysfunction and worse overall outcome after SOT [56]. Novel therapeutic agents are in great demand to further push the development of HTx.

The effect of RLX on the heart is widely studied showing that RLX is increasing coronary flow or remodeling post-infarction cardiac tissue in acute or chronic heart failure [57]. Although in a recent randomized clinical trial RLX showed no beneficial effect when treating acute heart failure, Dschietzig argues that there is a need to further investigate its effects as the trial had flaws [58,59]. In our review, we only included studies that would investigate the benefit of RLX in HTx but no studies that would directly examine the effect of RLX in HTx could be identified. However 5 experimental animal studies that showed the beneficial RLX effect in reducing IRI in heart [39,40,41,42,43] were identified.

Valle Raleigh et al. conducted a study in wild-type and endothelial nitric oxide synthase (eNOS) knockout mice model using RLX as possible agent reducing IRI after myocardial infarction [39]. Mice were pretreated with RLX (10 µg/kg) subcutaneously 1 h before ischemia or 5 m before reperfusion. The heart ischemia was induced for 30 m by occlusion of the left descending coronary artery, followed by reperfusion for 24 h. The results of this study showed that in both RLX treated groups, the overall survival rate was improved compared to controls. In addition, RLX also reduced myocardial infarct size, preserved left ventricle fractional shortening and end-systolic diameter. Administered at reperfusion, RLX reduced activity of caspase-1 in the heart of wild-type mice. These results were not observed in eNOS knockout mice, confirming a previously reported finding that RLX attenuates IR-induced heart injury via NO-dependent pathways. Interestingly, the administration time of RLX did not influence the effect, as the results in both pretreatment and reperfusion groups did not differ. Another study was performed by Perna et al. using a swine model in which myocardial infarction was induced by ligating the left anterior descending coronary artery and causing ischemia for 30 m with a followed reperfusion of 3 h [40]. In this study, RLX was administered at three different doses of 1.25, 2.5, and 5 μg/kg as a continuous infusion through the right atrial catheter for 20 m, starting at the time of reperfusion. The positive RLX effect was dose dependent, and the dose of 5 μg/kg showed the best myocardial protection. The administration of RLX lowered IRI markers, such as MPO and MDA levels, and the tissue calcium overload. RLX reduced apoptosis in cardiomyocytes and decreased the ultrastructural signs of cardiomyocyte damage. In the 5 μg/kg of RLX group almost no ultrastructural changes after IRI were observed.

Most of the articles emphasize that RLX acts by inducing NO synthase, and interestingly, Masini et al. prove that NO synthase can have dual roles in the myocardium [41]. In their study, RLX or a selective inhibitor of inducible NO synthase were used 30 m before ischemia in an experimental rat model. Unexpectedly, the results revealed that IRI can be reduced not only by increasing NO with the help of RLX but also by reduction applying a selective NO synthase inhibitor. It seems that the protection against IRI can be achieved by controlling the amount of NO in the tissue.

Masini et al. conducted another study in a perfused guinea pig heart model in which ischemia and reperfusion lasted for 20 m each, and RLX was added to the perfusate at the dose of 30 ng/mL [42]. RLX showed its effect by reducing MDA, calcium and histamine content compared to controls. Moreover, it reduced histological changes caused by IRI and the light transmittance across mast cells showing decreased content of secretory granules. One of the most important findings of this study was that RLX retained unchanged heart contractility function as compared to baseline values. In their other similar study, it was shown that RLX reduces myocardial IRI by stimulating the NO production and increasing coronary flow [43].

Overall, RLX in all studies showed a protective effect in non-transplant IRI animal models. There are concerns how these studies would be reproduced with human subjects, as the results from a recent randomized clinical trial examining the effect of RLX in acute heart failure were disappointing [58].

### 3.5. Possible Limitations of the Studies

Several possible limitations arise from these studies. Firstly, only one study examines the role of RLX in a transplantation setting, while all the others analyze the effect of RLX in reducing IRI in a non-transplant setting, such as isolated organ models or cold organ storage. Secondly, all included studies investigate RLX effect only on healthy organs. Up to now, no studies on extended criteria donor (ECD) organs, comprising among others organs after prolonged ischemia, from donors after circulatory death (DCD) and organs affected by hepatic steatosis were performed. As Lee et al. showed, RLX could be beneficial in attenuating hepatic steatosis and fibrosis in mice with non-alcoholic fatty liver disease [60]. Theoretically, this finding could be used for improving ECD organ quality prior to transplantation; however, this still needs to be elucidated and experimental and clinical studies are needed to prove the concept. Lastly, there are some methodological differences between the studies, which should also be taken into account. For instance, RLX was administered at different concentrations and routes; the ischemia and reperfusion times varied. Surprisingly, despite these differences, all included studies showed promising results.

## 4. Conclusions

Although RLX in a SOT setting is not widely studied, our review of currently available literature demonstrates a positive RLX effect in this field. Results from the studies show that it attenuates histological damage, inhibits apoptosis and reduces biochemical markers associated with IRI. Most importantly, RLX preserves the overall function of the organ and improves the overall survival. All these findings support a rationale to conduct first clinical studies to apply RLX as a potential substance in order to maintain organ integrity and ameliorate transplantation success after IRI.

## Figures and Tables

**Table 1:** Relaxin application in preclinical trials.

Author	Organ	Experimental Animal	Intervention	RLX Application	Effect of RLX
Kageyama et al. [30,31]	Liver	Mice	Liver transplantation after 18 h of cold storage in UW solution without RLX.	rhRLX dose of 5 µg/kg intravenously at the onset of reperfusion.	Improved overall survival. Decreased number of apoptotic cells. Attenuated sinusoidal congestion, edema/vacuolization, and hepatocellular necrosis. Acts through hepatocyte GR receptors. Induces Notch 1 signaling pathway.
Boehnert et al. [32,33]	Liver	Rats	Isolated healthy liver perfusion model after 5 h of cold or warm ischemia in HTK solution with or without RLX.	64 ng/mL of rhRLX was used in the preservation solution.	Protective effect against reperfusion injury indicated by reduced levels of MDA and MPO.
Boehnert et al. [34]	Liver	Rats	Isolated healthy liver perfusion model after 3.5 h of cold or warm ischemia in UW solution with or without RLX.	32 ng/mL of rhRLX was used in the reperfusion solution and 64 ng/mL of RLX in the preservation solution.	Protective effect against reperfusion injury indicated by reduced levels of MDA and MPO.
Yoshida et al. [35]	Kidney	Rats	Kidney IRI model with 45 m of ischemia and 24 h of reperfusion.	500 ng/h of porcine RLX 2 h before onset of reperfusion using an osmotic minipump under the skin.	Improved renal function. Attenuated post-IR increase in TNF-alpha. Ameliorated histological damage after IR. Reduced the number of apoptotic cells. Reduced the expression of TNFR1 mRNA in the kidney.
Collino et al. [36]	Kidney	Rats	Kidney IRI model with 60 m of ischemia and 6 h of reperfusion.	5 µg/kg of rhRLX intravenously at the onset of reperfusion and again 3 h after reperfusion.	Increased creatinine clearance. Attenuated renal cell damage after IR. Abolished IR-induced reduction in MnSOD and CuZnSOD. Reduced the activity of MPO. Reduced expression of ICAM-1. Prevented I/R-induced rise in IL-1b, IL-18 and TNF-alfa, levels.
Alexiou et al. [37]	Lung	Rats	Isolated healthy lung perfusion model using Krebs–Henseleit solution. Lungs exposed to 60 m of ischemia and 60 m of reperfusion.	5 nmol/L of rhRLX in the perfusion solution.	Reduced biochemical and morphological markers of pulmonary injury. Reduced the activity of NE and MPO. Decreased the production of MDA. Inhibited production of ET-1 from endothelial cells.
Alexiou et al. [38]	Lung	Rats	Isolated healthy lung perfusion model using Krebs–Henseleit solution. Lungs exposed to 90 m of ischemia and 90 m of reperfusion.	5 nmol/L of rhRLX in the perfusion solution.	Protective effect on IR-induced lung injury via early and moderate iNOS induction, dependent on balanced ERK-1/2 and PI3K stimulation.
Valle Raleigh et al. [39]	Heart	Mice (C57BL and eNOS knockout).	Heart IRI model with 30 m of ischemia and 24 h of reperfusion.	10 µg/kg of rhRLX subcutaneously 60 m before IRI or 5 m before the onset of reperfusion.	Improved survival after IRI. Reduced myocardial infarct size. Preserved left ventricle fractional shortening and end-systolic diameter. Attenuated caspase-1 activity in the heart after IRI.
Perna et al. [40]	Heart	Pigs	Heart IRI model with 30 m of ischemia and 3 h of reperfusion.	1.25; 2.5; or 5 μg/kg of rhRLX given upon the onset of reperfusion by a continuous infusion through the right atrial catheter.	5 μg/kg of RLX afforded the best myocardial protection. Reduced MPO and MDA, tissue calcium overload. Reduced apoptosis in cardiomyocytes. Decreased ultrastructural signs of cardiomyocyte damage. Improved the contractile performance of the heart.
Masini et al. [41]	Heart	Rats	Heart IRI model with 30 m of ischemia and 60 m of reperfusion	100 ng of porcine RLX dissolved in 500 μL of saline injected intravenously 30 m before ischemia.	Reduced MPO and MDA, tissue calcium overload in the left ventricle.
Masini et al. [42]	Heart	Guinea pigs	Isolated healthy heart perfusion model using a modified Tyrode solution supplemented by a IRI model with 20 m of ischemia and 20 m of reperfusion.	30 ng/mL of porcine RLX in the perfusion solution	Reduced ischemia-reperfusion induced increase of MDA. Reduced the calcium content. Retained unchanged histamine amounts compared to control. Reduced the light transmittance across mast cells showing decreased content of secretory granules. Reduced histological changes caused by IRI. Retained the heart contractility unchanged as compared to basal mean value.
Masini et al. [43]	Heart	Guinea pigs	Isolated healthy heart perfusion model using a modified Tyrode solution supplemented by a IRI model with 20 m of ischemia and 20 m of reperfusion. IRI model with 30 m of ischemia and 60 m of reperfusion was used.	30 ng/mL of porcine RLX in the perfusion solution In the second IRI model 100 ng of porcine RLX injected intravenously 30 m before ischemia.	Increased the amount of NO_2_ in the perfusates. Increased coronary flow through stimulation of NO production. Reduced leukocytes infiltration and lipid peroxidation. Decreased IR-induced release of histamine and LDH.

rhRLX—recombinant human relaxin; UW—University of Wisconsin; SOD—superoxide dismutase; GR—glucocorticoid receptor; MDA—malondialdehyde; MPO—myeloperoxidase; NE—neutrophil elastase; IRI—ischemia-reperfusion injury.

## Supplementary Materials

Supplementary materials can be found at https://www.mdpi.com/1422-0067/21/2/631/s1.

## Abbreviations

SOT	Solid organ transplantation
IRI	Ischemia reperfusion injury
RLX	Relaxin
ECD	Extended criteria donors
RXFP1	Relaxin family peptide receptor 1
MeSH	Medical Subject Headings
rhRLX	Recombinant human relaxin
UW	University of Wisconsin
SOD	Superoxide dismutase
GR	Glucocorticoid receptor
MDA	Malondialdehyde
MPO	Myeloperoxidase
NE	Neutrophil elastase
GODT	Global Observatory on Donation and Transplantation
LTx	Liver transplantation
KTx	Kidney transplantation
HTx	Heart transplantation
LuTx	Lung transplantation
ERK1/2	Extracellular signal-regulated kinase-1/2
PI3K	Phosphatidylinositol-3 kinase
iNOS	Inducible nitric oxide synthase
eNOS	Endothelial nitric oxide synthase
ROS	Reactive oxygen species
MPTP	Mitochondrial permeability transition pore
ET-1	Endothelin-1
