# Supplementary material for: Relaxin Positively Influences Ischemia—Reperfusion Injury in Solid Organ Transplantation: A Comprehensive Review
Source: Int J Mol Sci. 2020 Jan 17;21(2):631. doi: 10.3390/ijms21020631 (PMC7013572; doi:10.3390/ijms21020631)
Supplement: Supplementary file 1 [file ijms-21-00631-s001.zip › ijms-681260-suppl 2/Supp_material_Flowchart.docx]

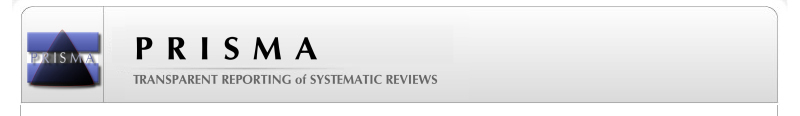
**PRISMA 2009 Flow Diagram**

`

Full-text articles excluded, with reasons
(n = 7)

Conference paper (7)

Review article (1)

Non-transplant or Non-IRI setting (2)

Full-text articles assessed for eligibility
(n = 24)

Studies included in the review
(n = 14)

Records excluded
(n = 191)

Records screened
(n = 215)

Records after duplicates removed
(n = 215)

Additional records identified through other sources
(n = 2)

## Identification

## Eligibility

## Included

## Screening

Records identified through database searching
(n = 655)
