# Supplementary material for: Relaxin Positively Influences Ischemia—Reperfusion Injury in Solid Organ Transplantation: A Comprehensive Review
Source: Int J Mol Sci. 2020 Jan 17;21(2):631. doi: 10.3390/ijms21020631 (PMC7013572; doi:10.3390/ijms21020631)
Supplement: Supplementary file 1 [file ijms-21-00631-s001.zip › ijms-681260-suppl 2/Supp_material_Search_strategy.docx]

Full database specific search strategies:

SEARCH STRATEGY, PUBMED:

"Organ Transplantation"[MeSH Terms] OR "Transplantation"[MeSH Terms] OR (("Organ"[All Fields] AND "transplant"[All Fields]) OR "Organ transplant"[All Fields]) OR (("Organ"[All Fields] AND "transplantation"[All Fields]) OR "Organ transplantation"[All Fields]) OR (("Liver"[All Fields] AND "transplantation"[All Fields]) OR "liver transplantation"[All Fields]) OR (("kidney"[All Fields] AND "transplantation"[All Fields]) OR "kidney transplantation"[All Fields]) OR (("uterus"[All Fields] AND "transplantation"[All Fields]) OR "uterus transplantation"[All Fields]) OR (("heart"[All Fields] AND "transplantation"[All Fields]) OR "heart transplantation"[All Fields]) OR (("lung"[All Fields] AND "transplantation"[All Fields]) OR "lung transplantation"[All Fields]) OR (("pancreas"[All Fields] AND "transplantation"[All Fields]) OR "pancreas transplantation"[All Fields]) OR "Organ Preservation Solutions"[MeSH Terms] OR "Perfusion"[MeSH Terms] OR "Perfusion"[All Fields] OR (("Perfusion"[All Fields] AND "machine"[All Fields]) OR "Perfusion machine"[All Fields]) OR "Reperfusion Injury"[MeSH Terms] OR (("Hypoxia"[All Fields] AND "reoxygenation"[All Fields]) OR "hypoxia-reoxygenation"[All Fields]) OR (("Reperfusion"[All Fields] AND "Injury"[All Fields]) OR "Reperfusion Injury"[All Fields]) OR (("Ischemia"[All Fields] AND "Reperfusion"[All Fields] AND "Injury"[All Fields]) OR "Ischemia Reperfusion Injury"[All Fields]) OR (("Cold"[All Fields] AND "Storage"[All Fields]) OR "Cold Storage"[All Fields]) OR (("Cold"[All Fields] AND "ischemia"[All Fields]) OR "Cold ischemia"[All Fields]) OR "reperfusion"[All Fields] OR "ischemia"[All Fields] OR "ischaemia"[All Fields] OR (("ischemic"[All Fields] AND "reperfusion"[All Fields]) OR "ischemic reperfusion"[All Fields]) OR (("ischaemic"[All Fields] AND "reperfusion"[All Fields]) OR "ischaemic reperfusion"[All Fields]) **AND** ("Relaxin"[MeSH Terms] OR "Relaxin"[All Fields] OR "Serelaxin"[All Fields] OR "Recombinant human relaxin"[All Fields])

**Language:** English

**Time span:** All years

SEARCH STRATEGY, WEB OF SCIENCE:

TS=("Organ Transplantation" OR "Transplantation" OR "Organ transplant" OR "liver transplantation" OR "uterus transplantation" OR "kidney transplantation" OR "heart transplantation" OR "lung transplantation" OR "pancreas transplantation" OR "Organ Preservation Solutions" OR "Perfusion" OR "Perfusion machine" OR "Reperfusion Injury" OR "Ischemia Reperfusion Injury" OR "Cold Storage" OR "Cold ischemia" OR "hypoxia-reoxygenation" OR "reperfusion" OR "ischemia" OR "ischaemia" OR "ischemic reperfusion" OR "ischaemic reperfusion") **AND** TS=("Relaxin" OR "Serelaxin" OR "Recombinant human relaxin")

**Language:** English

**Time span:** All years

SEARCH STRATEGY, EMBASE:

("Organ Transplantation" OR "Transplantation" OR "Organ transplant" OR "liver transplantation" OR "uterus transplantation" OR "kidney transplantation" OR "heart transplantation" OR "lung transplantation" OR "pancreas transplantation" OR "Organ Preservation Solutions" OR "Perfusion" OR "Perfusion machine" OR "Reperfusion Injury" OR "Ischemia Reperfusion Injury" OR "Cold Storage" OR "Cold ischemia" OR "hypoxia-reoxygenation" OR "reperfusion" OR "ischemia" OR "ischaemia" OR "ischemic reperfusion" OR "ischaemic reperfusion") **AND** ("Relaxin" OR "Serelaxin" OR "Recombinant human relaxin")

**Language:** English

**Time span:** All years

SEARCH STRATEGY, ClinicalTrials.gov:

"Relaxin" OR "Serelaxin"

**Language:** English

**Time span:** All years
